# Stably maintained microtubules protect dopamine neurons and alleviate depression-like behavior after intracerebral hemorrhage

**DOI:** 10.1038/s41598-018-31056-7

**Published:** 2018-08-23

**Authors:** Yang Yang, Kaiyuan Zhang, Jun Zhong, Ju Wang, Zhongyuan Yu, Xuejiao Lei, Xuezhu Chen, Yulian Quan, Jishu Xian, Yujie Chen, Xin Liu, Hua Feng, Liang Tan

**Affiliations:** 1 Department of Neurosurgery, Southwest Hospital, Third Military Medical University (Army Medical University), Chongqing, 29 Gaotanyan Street, 400038 China; 2 Battalion 3 of Cadet Brigade, Third Military Medical University (Army Medical University), Chongqing, 29 Gaotanyan Street, 400038 China

**Keywords:** Depression, Stroke

## Abstract

Mesolimbic dopamine (DA) system lesion plays a key role in the pathophysiology of depression, and our previous study demonstrated that reduced microtubule (MT) stability aggravated nigrostriatal pathway impairment after intracerebral hemorrhage (ICH). This study aimed to further investigate the occurrence regularity of depression-like behavior after ICH and determine whether maintaining MT stabilization could protect DA neurons in ventral tegmental area (VTA) and alleviate depression-like behavior after ICH. An intrastriatal injection of 20 μl of autologous blood or MT depolymerization reagent nocodazole (Noco) was used to mimic the pathology of ICH model in mice. The concentration of DA, number of DA neurons and acetylated α-tubulin (a marker for stable MT) in VTA were checked, and depression-related behavior tests were performed after ICH. A MT-stabilizing agent, epothilone B (EpoB), was administered to explore the effects of MT stabilization on DA neurons and depression-like behavior after ICH. The results showed that obvious depression-like behavior occurred at 7, 14, and 28 days (*P* < 0.01) after ICH. These time-points were related to significant decreases in the concentration of DA (*P* < 0.01) and number of DA neurons (*P* < 0.01) in VTA. Moreover, The decrease of acetylated α-tubulin expression after ICH and Noco injection contributed to DA neurons’ impairment in VTA, and Noco injecton also aggravate ICH-induced depression-like behaviors and DA neurons’ injury. Furthermore, EpoB treatment significantly ameliorated ICH and Noco-induced depression-like behaviors (*P* < 0.05) and increased the concentration of DA (*P* < 0.05) and number of DA neurons (*P* < 0.05) in VTA by increasing the level of acetylated α-tubulin. The results indicate that EpoB can protect DA neurons by enhancing MT stability, and alleviate post-ICH depressive behaviors. This MT-targeted therapeutic strategy shows promise as a bench-to-bedside translational method for treating depression after ICH.

## Introduction

Post-stroke depression is the most frequent psychiatric disorder following stroke, and it is independently associated with increased mortality, morbidity and poorer survival-related functional outcomes^1–3^. Intracerebral hemorrhage (ICH) is a severe type of stroke, and depression has been reported to be present in 20% of ICH survivors^4,5^. However, even though there are many studies have examined depression after ICH in clinical and basic experiments, the etiological factors that lead to post-ICH depression remain largely unknown. Therefore, understanding the specific pathogenesis of depression after ICH is vital for developing effective therapeutic strategies aimed at etiological factors.

Dopamine (DA) plays a vital and important role in motor control, reward-motivated behavior, and depression ^6–8^. Emerging evidence suggests that impairment of DA neurons in ventral tegmental area (VTA) may underlie the pathophysiology of several psychiatric disorders, including schizophrenia and depression^6,9^. Recent studies show that rats exposed to chronic cold or unpredictable chronic mild stressors (UCMS) have been shown to exhibit extended decreases (by approximately 50%) in VTA DA neuron population^10,11^, and activation of VTA DA neuron firing can reverse the depressive state. Our previous study also demonstrated that DA neuronal apoptosis and decreased DA levels contributed to depression-like behavior after traumatic brain injury^12^, indicating that protecting DA neurons and increasing DA concentrations could alleviate depression-like behavior after ICH. However, the mechanism underlying secondary damage of DA neuron in impaired remote region at VTA after ICH remain unclear.

As an important component of the cytoskeleton, microtubule (MT) participates in different cellular processes, including mechanical support, cell motility, the organization of the cytoplasm, and secretory vesicle transport^13^. Recent studies have demonstrated that midbrain DA neurons are particularly vulnerable to microtubule disruptions^14^. Furthermore, our previous research also demonstrated that striatal hemorrhage leads to motor dysfunction and DA neuron degeneration in the substantia nigra (SN) as a result of the reduced stability of MT^15^. These data indicated that ICH-induced MT destabilization might contribute to DA neuronal injury and that MT-targeted therapeutic strategies could alleviate DA neuronal injury. This presents a promising translational perspective for treating post-ICH depression.

In this study, we aimed to further explore the potential mechanisms underlying depression after ICH and identify a feasible treatment approach for depression post-ICH. It’s hypothesized that ICH-induced reduction of MT stability led to DA neuronal injury and decrease of DA levels in VTA, which may contribute to the depression-like behavior following ICH. The administration of the MT-stabilizing agent, epothilone B (EpoB), could alleviate DA neuronal impairment, increase DA concentrations and improve depression-like behavior by stabilizing MT.

## Results

### Depression-like behavior appeared at 7–28 days after ICH

**Figure 1:**
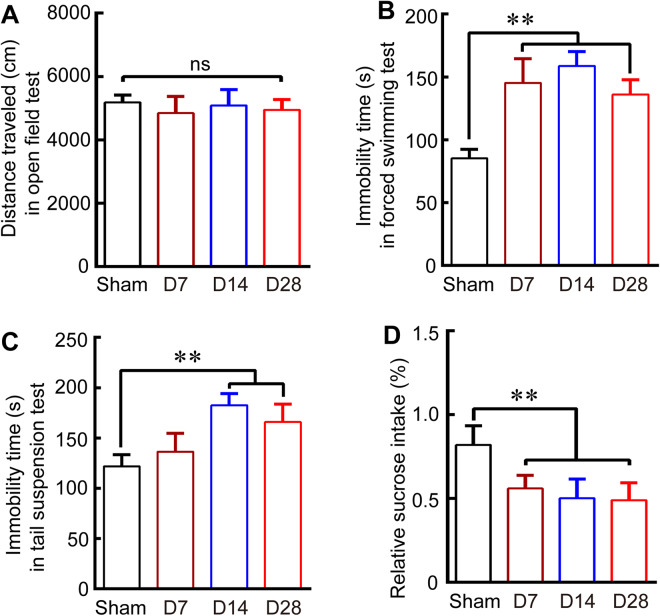
Depression-like behavior was detected after ICH in the sucrose preference test, forced swimming test (FST) and tail suspension test (TST). (**A**) Quantitative data for total distance travelled in the open-field test over a period of 10 min at 7, 14 and 28 days after ICH. (**B**-**C**) Quantitative data for immobility time(s) in the FST (A) and TST (B) in mice in the sham group and at 7, 14, and 28 days post-ICH. (**D**) Quantitative data for relative sucrose intake (%) in the sham group and at 7, 14, and 28 days post-ICH. Data are shown as the means ± SEM (at least n = 8 for the behavior tests in each group; **P < 0.01 versus the Sham group; ns = not significant).

We found that there was no significant difference in total travelled distance between sham group and ICH group at the day of 7, 14 and 28 (Fig. 1A; *P* > 0.05). Next, we employed the forced swimming test (FST), tail suspension test (TST) and sucrose preference test to measure depression-like behaviors after ICH. We found that ICH group exhibited a significant increase in immobility time (s) in the FST at 7, 14, and 28 days compared with the sham group (Fig. 1B; *P* < 0.01). In the TST test, ICH led to only an apparent increase over the sham group in immobility time (s) at 14 and 28 days (Fig. 1C; *P* < 0.01). In addition, relative sucrose intake (%) in the sucrose preference was significantly lower at 7, 14, and 28 days in the ICH group than in the sham group (Fig. 1D; *P* < 0.01).

### Reduced MT stability was associated with damage of DA neurons and lower DA concentrations after ICH

**Figure 2:**
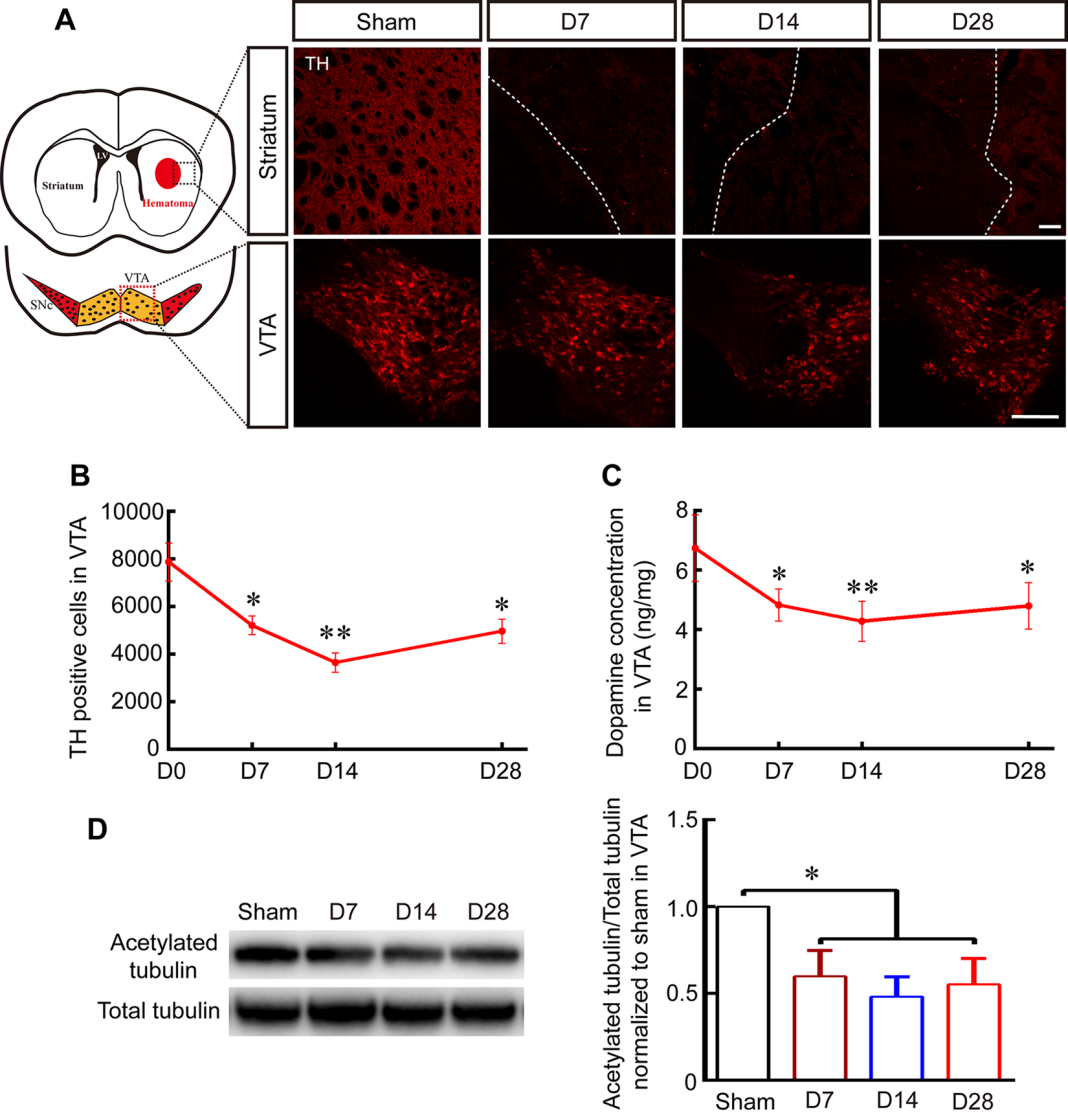
ICH-induced MT depolymerization contributed to impairments in DA neurons’ numbers and DA concentrations. (**A**) Representative pictures of TH-labelled DA fibers and neurons in the striatum and VTA in the sham group and at 7, 14, and 28 days post-ICH (Scale bar: 100 µm; red dotted grid indicates the observation area, and white dotted lines distinguish the hematoma area and the surrounding unaffected tissues). (**B**) The number of TH-positive cells in the VTA was evaluated in a stereological analysis at 7, 14, and 28 days post-ICH and in the sham group (replaced by 0 day). (**C**) The dopamine concentration was measured in VTA using HPLC-ECD at 7, 14, and 28 days after ICH and in the sham group (replaced by 0 day). (**D**) Representative Western blot images and quantitative data for acetylated α-tubulin and total tubulin expression in the VTA at 7, 14, and 28 days post-ICH and in the sham group (Full-length blots/gels are presented in Supplementary Figure S1). Data are shown as the means ± SEM (at least n = 5 for each group; *P < 0.05 and **P < 0.01 versus sham group). SNc = substantia nigra pars compacta; LV = lateral ventricle; TH = tyrosine hydroxylase.

We next investigated the pathomorphological changes of DA neurons in VTA and fibers in striatum. Pathological lesions in DA fibers and neurons in the striatum and VTA were analyzed from 7–28 days after ICH. TH-positive DA fibers and neurons were lower in the striatum and VTA at 7, 14, and 28 days in the ICH group than in the sham group (Fig. 2A). The stereological analysis results showed that the number of TH-positive cells in the VTA was significantly lower at 7, 14, and 28 days after ICH than in the sham group (Fig. 2B; *P* < 0.05). Moreover, the DA concentration was also significantly lower in VTA following ICH (Fig. 2C; *P* < 0.05), in accordance with our previous study, and these changes contributed to the development of depression-like behaviors. Acetylation is generally thought to occur on stable MT assemblies, and acetylated α-tubulin is a common marker of stabilized MT. Interestingly, the level of acetylated α-tubulin was also significantly lower at 7, 14, and 28 days after ICH than in the sham group (Fig. 2D; *P* < 0.05), indicating that unstable and depolymerized MT may contribute to the lower numbers of DA neurons and DA concentrations observed after ICH.

### EpoB administration ameliorated impairments in DA neurons and DA concentrations by promoting MT stabilization

**Figure 3:**
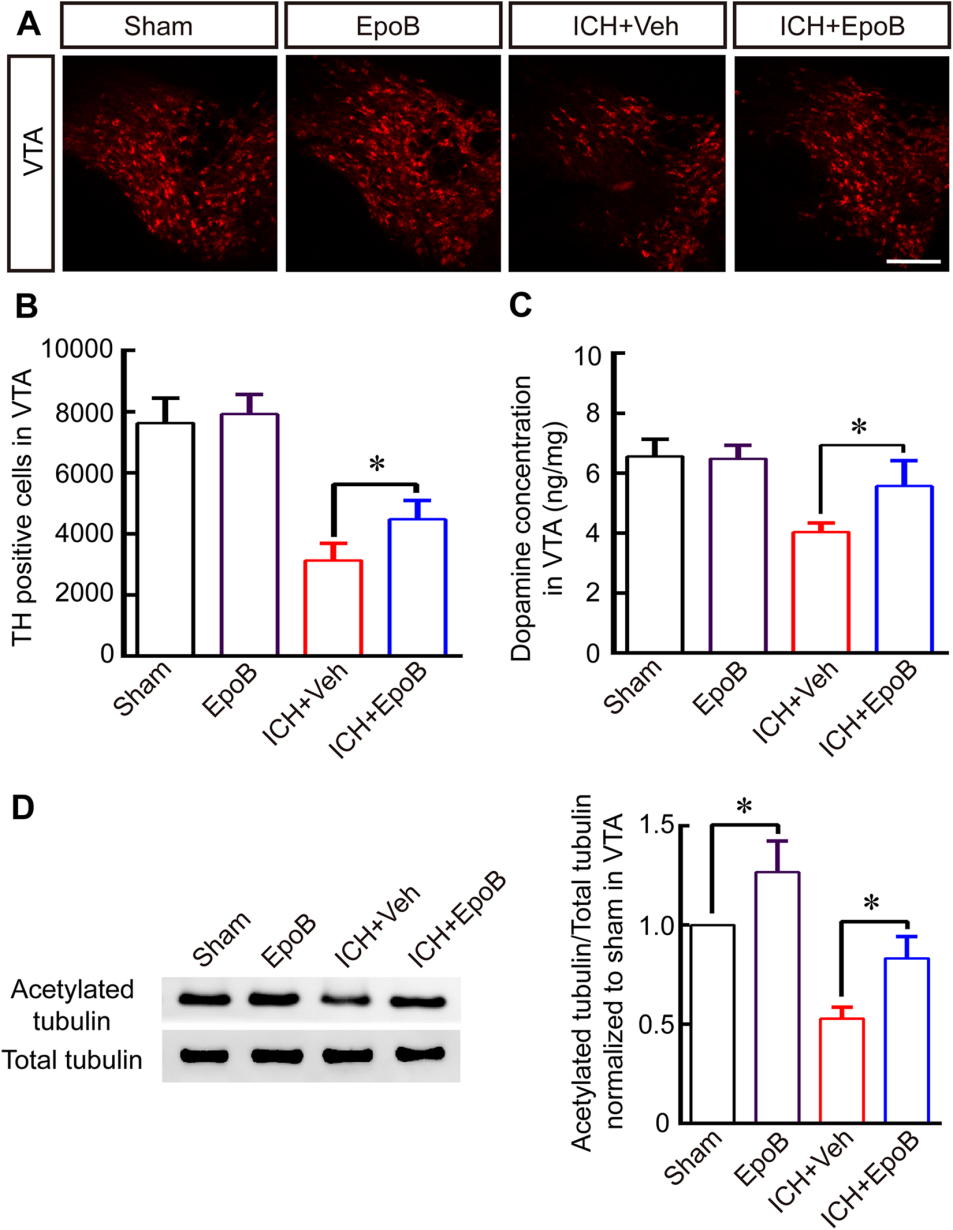
EpoB treatment rescues DA neurons and DA concentrations by promoting MT stabilization after ICH. (**A**) Representative immunofluorescence staining for TH in VTA in the Sham, EpoB, ICH + Veh and ICH + EpoB groups at 14 days after ICH (Scale bar = 100 μm). (**B**-**C**) Quantitative data for TH-positive cell numbers (**B**) and dopamine concentrations (**C**) in the VTA in the Sham, EpoB, ICH + Veh and ICH + EpoB groups at 14 days post-ICH. (**D**) Representative Western blot images and quantitative data for acetylated α-tubulin and total tubulin expression in the VTA in the four groups at 14 days post-ICH (Full-length blots/gels are presented in Supplementary Figure S2). Data are shown as the means ± SEM (at least n = 5 for each group; *P < 0.05).

Next, we studied the EpoB protective function of VTA DA neurons and DA concentration after ICH. We found that both the most obvious depression-like behaviors and the lowest number of DA neurons were observed at 14 days after ICH. We therefore selected 14 days after ICH to study the protective function of EpoB. Here, we revealed that EpoB administration apparently increased the level of acetylated α-tubulin in the VTA (Fig. 3D; *P* < 0.05), as detected using Western blotting, compared to what was observed in the ICH + Veh group at 14 days after ICH. Fortunately, EpoB treatment significantly increased the number of TH-positive DA neurons in VTA (Fig. 3A-B; *P* < 0.05) compared to what was observed in the ICH + Veh group at 14 days after ICH. Moreover, the concentration of DA was also higher in the ICH + EpoB group than in the ICH + Veh group (Fig. 3C; *P* < 0.05) at 14 days after ICH. These results indicated that EpoB might protect DA concentrations and DA neurons by maintaining MT stabilization in DA neurons.

### Noco-induced MT depolymerization aggravated ICH injury and EpoB treatment protected VTA DA neurons injury against Noco-induced MT depolymerization

**Figure 4:**
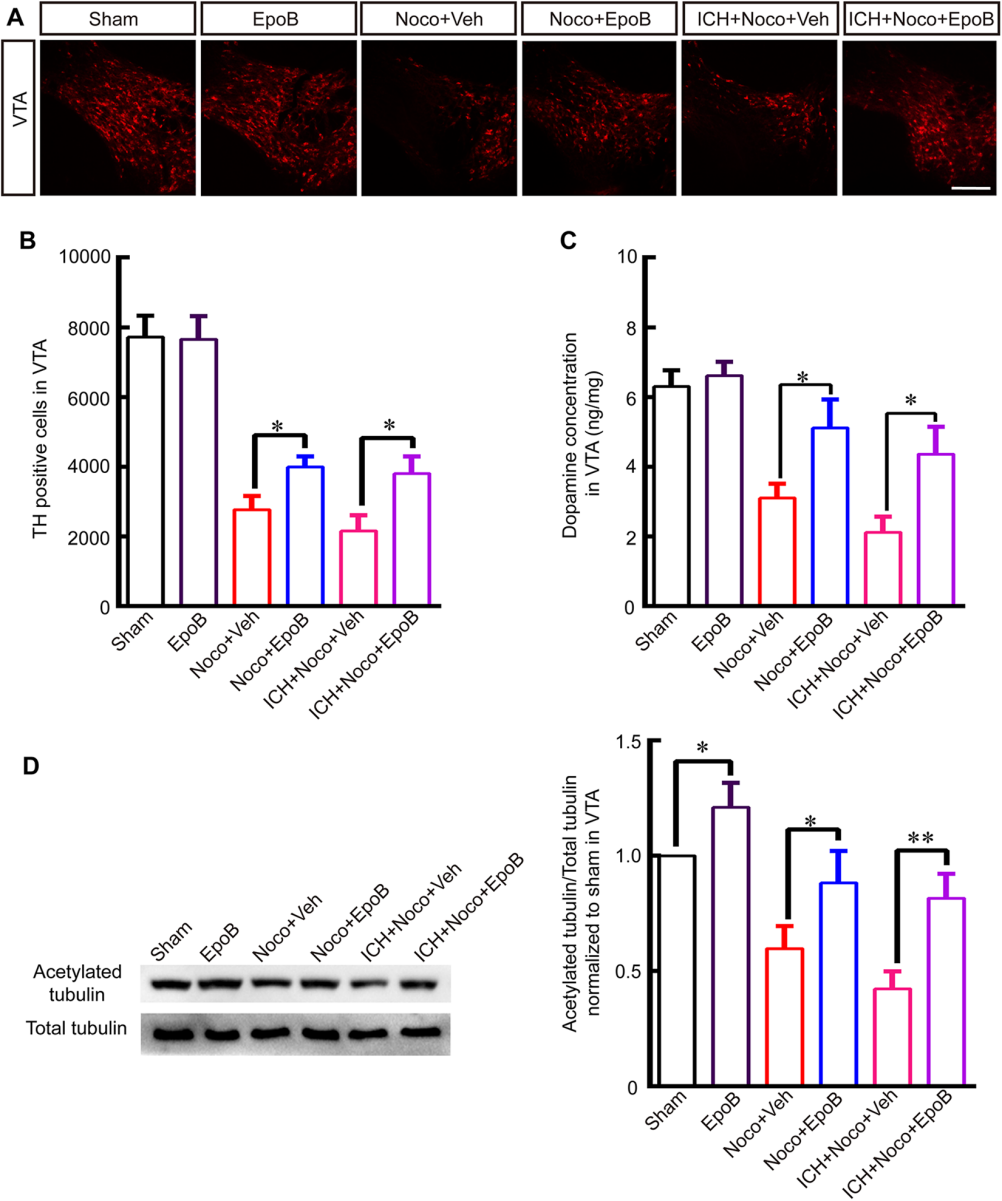
Noco-induced MT depolymerization aggravated ICH injury and EpoB treatment protected VTA DA neurons’ injury against Noco-induced MT depolymerization. (**A**) Representative immunofluorescence staining for TH in VTA in the Sham, EpoB, Noco + Veh, Noco + EpoB, ICH + Noco + Veh and ICH + Noco + EpoB groups at 14 days after Noco injection or ICH (Scale bar = 100 μm). (**B**-**C**) Quantitative data for TH-positive cell (**B**) and DA concentrations (**C**) in the VTA in the Sham, EpoB, Noco + Veh, Noco + EpoB, ICH + Noco + Veh and ICH + Noco + EpoB groups at 14 days after Noco injection or ICH. (**D**) Representative Western blot images and quantitative data for acetylated α-tubulin and total tubulin expression in the VTA in the six groups at 14 days after Noco injection or ICH (Full-length blots/gels are presented in Supplementary Figure S3). Data are shown as the means ± SEM (at least n = 5 for each group; *P < 0.05).

**Figure 5:**
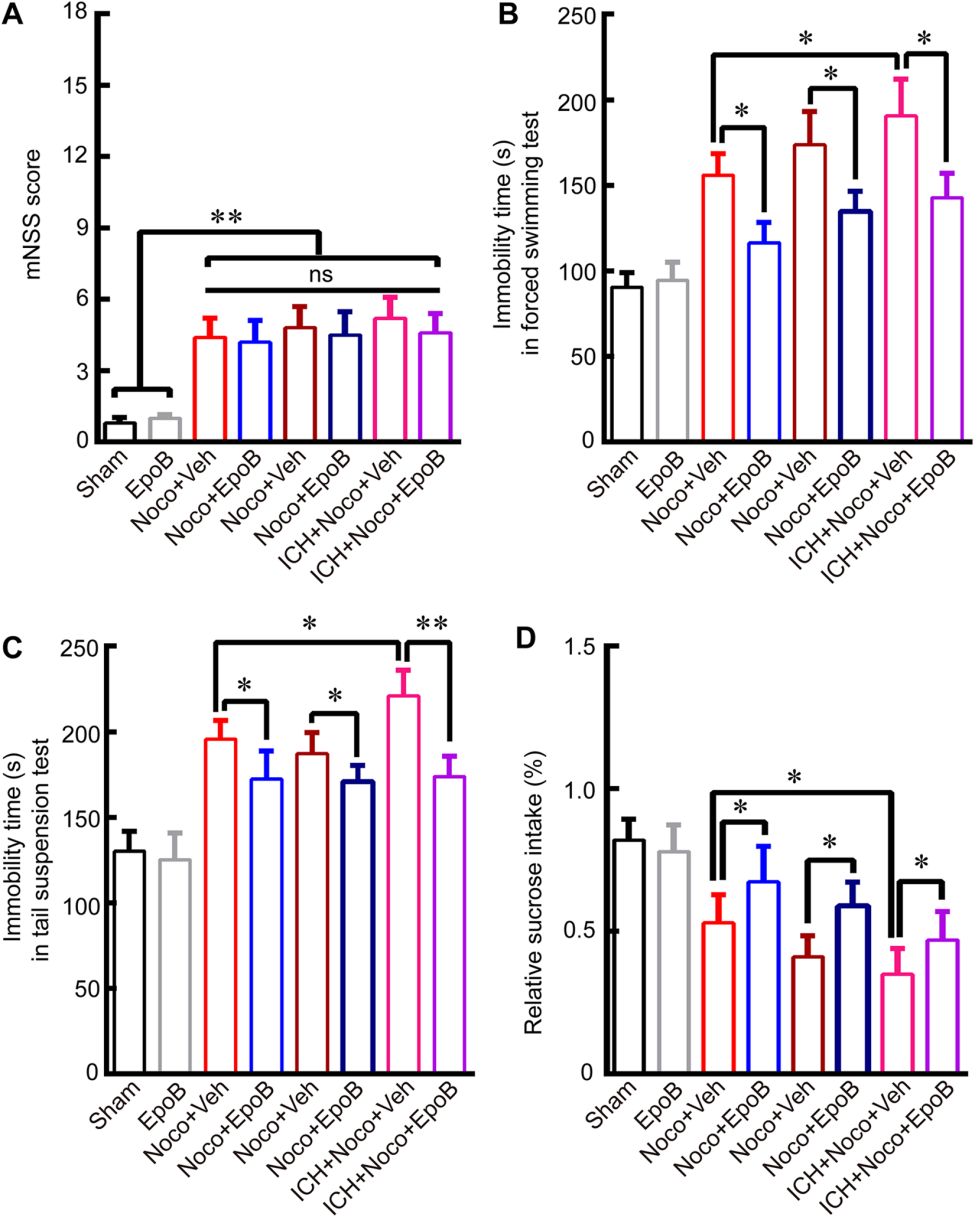
EpoB treatment alleviates depression-like behavior after ICH and Noco injection. (**A**) mNSS score at 14 days after ICH or Noco injection for all treatment groups. (**B**-**C**) Quantitative data for immobility time (s) in the FST (A) and TST (B) in mice in the eight groups at 14 days after ICH and Noco injection. (**D**) Quantitative data for relative sucrose intake (%) in the eight groups at 14 days post-ICH and Noco injection. Data are shown as the means ± SEM (at least n = 8 for the behavior tests in each group; *P < 0.05, **P < 0.01 and ns = not significant).

In this study, in order to ensure that MT depolymerization and instability were a pivotal process in VTA DA neurons impairment after ICH, we established model of intrastriatal injection of Noco with or without ICH. As a MT depolymerization reagent, Noco injection significantly decreased the level of acetylated α-tubulin (Fig. 4D), accompanied with obvious depression-like behaviors (Fig. 5B–D; *P* < 0.01), decreased DA neurons’ number and DA concentration (Fig. 4A–C; *P* < 0.05) in VTA at 14 days after injection. What’s more, Noco injection could aggravate the injury of VTA DA neurons after ICH by decreasing the DA neurons’ number and DA concentration in VTA area (Fig. 4A–C) and worsen the depression-like behaviors compared with ICH + Veh group (Fig. 5B–D; *P* < 0.05). Similarly, EpoB treatment significantly increased the the level of acetylated α-tubulin, the number of DA neurons and DA concentration (Fig. 4A–D; *P* < 0.05) in VTA at 14 days after injection compared with the levels in the Noco + Veh and ICH + Noco + Veh groups.

### EpoB administration alleviated depression-like behavior after ICH and Noco injection

After examining the protective effects of EpoB on DA neuronal morphology and DA concentrations, here we detected the protective effects of EpoB administration on depressive behaviors at 14 days after ICH and Noco injection. Firstly, in order to exclude the effects of motor deficits on depression behavioral tests, mNSS (including motor, sensory, reflex, and balance tests) was used for examining all the treatment groups. Interestingly, there were no significant difference among groups of ICH + Veh, ICH + EpoB, Noco + Veh, Noco + EpoB, ICH + Noco + Veh and ICH + Noco + EpoB in mNSS (Fig. 5A). However, EpoB treatment resulted in significant lower immobility time(s) on the FST and TST at 14 days after ICH and Noco injection than in the ICH + Veh, Noco + Veh and ICH + Noco + Veh groups (Fig. 5B,C; *P* < 0.05). Furthermore, the relative sucrose intake (%) on the sucrose preference was also significantly higher at 14 days in the ICH + EpoB, Noco + EpoB and ICH + Noco + EpoB groups than in the ICH + Veh, Noco + Veh and ICH + Noco + Veh groups (Fig. 5D; *P* < 0.05). These behavioral results indicated that maintaining MT stabilization with EpoB could alleviate depression-like behavior after ICH.

## Discussion

In the present study, our data showed that striatal hemorrhage induced MT instability contributed to severe damage of DA neurons in VTA, thereby significantly decreasing DA concentrations and inducing animals’ depression-like behaviors after ICH. The MT-stabilizing agent EpoB alleviated DA neuronal impairment, increased DA concentrations and ameliorated depression-like behaviors by promoting MT stabilization after ICH.

Depression is a debilitating disease characterized by various symptoms that include a depressed mood, anhedonia, hopelessness and reduced motivation^16,17^. The monoamine hypothesis, which states that the lack of monoamine neurotransmitters such as serotonin, DA and norepinephrine, causes depression symptoms, has dominated academia for the past several decades^18,19^. In particular, the DA system is unique among the brain’s modulatory systems, in that it has discrete projections to specific brain regions involved in motor behavior, cognition and emotion^6^. Accumulating studies suggested that disruptions in mesolimbic DA systems play a key role in the pathophysiology of depression^9^ and that DA precursors, DA agonists, DA reuptake inhibitors^20–22^ and the methods that activate DA neurons via optogenetic techniques^23^ have therapeutic efficacy in depression. Our previous study demonstrated that midbrain DA system disruptions led to depression-like behavior after traumatic brain injury (TBI)^12^. Here, we also found that ICH induced severe damage to the midbrain DA system by significantly decreasing the number of DA neurons and DA concentrations in VTA (Fig. 2), and that depression-like behaviors occurred from 7–28 days after ICH and were most obvious at 14 days after ICH (Fig. 1). Despite the numerous clinical and experimental studies mentioned above, the pathophysiological mechanisms underlying the development of depression after ICH or TBI remain far from clear.

Compared with other types of neurons, DA neurons in the VTA are more susceptible to primary and secondary lesion in both humans and animal models, but the features that confer this susceptibility in DA neurons remain incompletely understood^24,25^. Recent studies in environmental toxicology and molecular genetics have demonstrated that midbrain DA neurons and its fibers are particularly vulnerable to microtubule depolymerization^14^ and that Parkinsonism is a late-onset symptoms associated with the above injuries^26^. Furthermore, other studies have indicated that the striatal injection of the microtubule disruptors colchicine^14^ and nocodazole^15^ leads to the degeneration of DA neurons and its fibers in the SN as the result of a reduction in the stability of MT. In addition, emerging evidence suggests that the pathogenesis of depressive disorders is associated with neuronal abnormalities in brain MT function, including changes in α-tubulin isoforms^27–29^, and decrease of MT associated axonal transport proteins also contributed to stress-induced depressive behavior^30^. The aforementioned evidences indicate that MT destabilization and dysfunction might contribute to depression-like behavior by impairing VTA DA neurons after ICH, this pathophysiological mechanism probably related with DA neurons axonal transport barrier. Interestingly, our previous work revealed that hemorrhage in the striatum also led to the degeneration of DA fibers in the striatum and the bodies of DA neurons in the SN, and these effects were also related to a reduction in the stability of MT^15^. Our recent works revealed that there was a significant decrease in both number of DA neurons and the level of acetylated α-tubulin, a marker for stably maintained MT, in VTA at 7–28 days after ICH, indicating that ICH might result in lower DA concentration and depression-like behavior by reducing the stabilization of MT in DA neurons (Fig. 2). However, according to our data, the impaired DA level in VTA grew at day 28 (Fig. 2C), which is consistent with previous study^12^, which may be associated with residual DA neurons hyperfunction and increased DA release metabolism after brain injury^12,31^. What’s more, TH positives cells also increased at day 28 after ICH in VTA (Fig. 2B), which may be related with increased protein expression of TH or regeneration. What’s more, simple intrastriatal injection of MT depolymerization reagent Noco significantly led to depression-like behavior, and Noco injection also worsen the DA neurons’ injury and aggravate depression-like behaviors after ICH (Figs 4–5), this result powerfully proved that single reduction of MT stabilization might be an important mechanism during onset and progression of depression. Fortunately, the systemic administration of 1.5 mg/Kg MT stabilizer EpoB increased the the level of acetylated α-tubulin in VTA, significantly alleviated depression-like behaviors, and increased the DA concentration after ICH and Noco injection by promoting MT stabilization (Figs 3–5), consistent with previous studies showing that EpoB promoted axonal regeneration in Alzheimer’s disease^32^ and spinal cord injury^33,34^ by maintaining MT stabilization. In addition, the systemic administration of EpoB had no apparent side effects^15,34^, making EpoB a promising candidate for clinical testing.

There are still some shortcomings associated with our recent work, and these need to be accounted for in future studies. First, the observation period for depression-related behaviors in mice after ICH was relatively short, and the therapeutic time point of EpoB was only 14 days after ICH. Second, *in vitro* studies are needed to further determine the mechanism underlying how unstable or unpolymerized MT can lead to damage of DA neurons and DA concentration. Third, neural circuits play important roles in human emotions^35,36^, and lesion of DA concentrations in some emotion-related neural circuits (e.g., the Papez circuit), may lead to depression-like behavior after ICH^37^. We will further study the function of DA in the Papez and other circuits during depression occurrence after ICH.

In summary, a reduction in the stabilization of MT contributed to less number of DA neurons and lower DA concentration in VTA after striatal hemorrhage, and EpoB treatment alleviated lesions in the DA system and depression-like behaviors by maintaining MT stability and increasing the the level of acetylated α-tubulin. This MT-targeted therapeutic strategy shows promise as a bench-to-bedside translational strategy for treating depression-like behavior after ICH.

## Methods and Materials

### Animal

Adult male C57BL/6 mice (aged 8–10 weeks old, 20–25 g) were used in this study and provided by the Experimental Animal Center at the Third Military Medical University (permit number: Yu2017-0002; Chongqing, China). The mice were housed in a specific temperature-controlled room under a 12-h light/12-h dark cycle and provided free access to food and water throughout the experimental period.

### ICH Model

All experimental protocols were approved by the Ethics Committee of the Third Military Medical University and performed according to the health guide for the care and use of laboratory animals. The ICH model induction procedure utilized in this study has been described previously in detail^38,39^. Briefly, after the mice were anesthetized with 3% isoflurane (in mixed air and oxygen), they were placed onto a stereotaxic frame in a prone position. Then, a small cranial burr hole was made at a precise location (bregma coordinates: 0.8 mm anterior and 2 mm lateral to the midline) under stereotactic guidance using aseptic techniques, and 20 μl of autologous blood was injected 3 mm deep into the right basal ganglia at a rate of 2 μl/min using a sterile 33 G Hamilton syringe needle and a microinfusion pump (Harvard Apparatus, Holliston, MA). The mice were divided into the following three groups: Sham, EpoB, ICH + Vehicle (ICH + Veh) and ICH + EpoB. According to previous study^15,34^, the ICH mice received EpoB (1.5 mg/kg i.p. dissolved in dimethyl sulfoxide (DMSO) and saline at a ratio of 1:3) or an equal volume of vehicle 2 h after the ICH procedure. At 7, 14 and 28 days after surgery, the mice underwent depression-related behavioral tests and were then deeply anesthetized. The brains were then collected for morphological and biochemical experiments.

For the MT injury model, 10 μl of 100 µM MT destabilizing agent Noco (diluted into 5% DMSO in saline; Sigma-Aldrich, St. Louis, MO) or 5% DMSO alone was injected into the striatum using a sterile microsyringe. After 2 hours, the MT injury mice were intraperitoneally administered EpoB or vehicle. These mice were divided into two groups: Noco + Vehicle and Noco + EpoB. At 14 days after surgery, the mice underwent depression-related behavioral tests. The mice were then deeply anesthetized, and the brains were collected for morphological and biochemical experiment. What’s more, to explore whether Noco will worsen the injury of mice after ICH, ICH + Noco + Vehicle and ICH + Noco + EpoB groups also created as above description (To avoid the effect of Noco on autologous blood, 5 μl of 100 µM Noco was injected into the striatum at 2 days after ICH). At 14 days after surgery, the mice underwent depression-related behavioral tests and morphological and biochemical experiments.

Exclusive criterion: At 7 days after ICH or Noco injection, the mice were evaluated by the modified Neurological Severity Scores (mNSS) according to previous study^40,41^. mNSS was a composite of motor, sensory, reflex, and balance tests, and graded on a scale of 0 to 18 (normal score, 0; maximal deficit score, 18). One point is awarded for the inability to perform the tasks or for the lack of a tested reflex; 13 to 18 indicates severe injury; 7 to 12, moderate injury; 1 to 6, mild injury. The mice with severe motor deficiency (mNSS:13 to 18 points) and mild injury (mNSS:1 to 6 points) were excluded.

### Immunofluorescence

The immunofluorescence method used here was described in our previous research study^42^. Thirty micrometer-thick cryostat sections were incubated with primary antibodies against rabbit anti-tyrosine hydroxylase (TH, 1:1000, Millipore, Temecula, CA, USA) in 1% BSA overnight at 4 °C. This is a specific marker of DA neurons. After the sections were washed with 0.01 M phosphate buffer, they were probed with the appropriate Cy3- and Alexa 488-conjugated secondary antibodies (1:500 for 4 h at room temperature (RT); Jackson ImmunoResearch, West Grove, PA, USA). Finally, the sections were counterstained with 4′,6-diamidino-2-phenylindole (DAPI) (Santa Cruz Biotechnology) and mounted in Vectashield medium (Vector Laboratories) before they were photographed using a Zeiss confocal microscope (Zeiss, LSM780).

### High-performance liquid chromatography-electrochemical detection analysis

The concentration of DA in VTA of the ICH mice was measured using high-performance liquid chromatography-electrochemical detection (HPLC-ECD), which was performed according to the methods described by Tan *et al*.^12^. The mobile phase was composed of 100 mM NaH_2_PO_4_ in HPLC-grade water, and 1% acetonitrile (pH = 3) was imported into the reverse phase column at a flow rate of 0.8 ml/min. The chromatographic data were exported and analyzed using ESA software (ESA, Inc., USA). The area under the curve (AUC) of a standard concentration of DA was used to compare the DA concentration in the samples.

### Western blot analysis

Western blot (WB) was performed as described by Xie *et al*.^43^. The brain tissues at the ipsilateral VTA were collected for extraction. After gel electrophoresis was performed, the proteins were transferred to membranes, and the membranes were incubated with the following primary antibodies at 4 °C overnight: rabbit anti-TH (1:1000, Millipore), rabbit anti-acetylated α-tubulin (acetyl K40, acetyl-tubulin, 1:1000; CST and Abcam), and mouse anti-α-tubulin (T-tubulin, 1:1000; Boster, Wuhan, China), which was used as a loading control. Then, the membranes were probed with specific horseradish peroxidase-conjugated secondary antibodies (1:1000, Beyotime biotechnology) for 3 h at RT. Finally, an enhanced chemiluminescence reagent kit (Millipore, Temecula, CA, USA) for Western blotting was used to visualize the immunoreactive bands, which were detected with a bioimaging system (VersaDoc MP 4000; Bio-Rad, Hercules, CA). Five animals from each group and at least three repetitions of each treatment condition were used for Western blot analyses.

### Behavior tests

Open field test was used to assesses locomotor and behavioral activity levels of mice after ICH, this test was measured using an open-field activity system (Biowill, Shanghai, China) and analyzed using Noldus EthoVision XT software (Noldus Information Technology Co., Ltd, Netherlands) as previous described^15^.

The forced swimming test (FST), tail suspension test (TST) and sucrose preference test were employed to quantify depression-like symptoms after ICH. The mice were acclimatized to the experimental room for at least 30 min prior to each test.

The FST was completed according to previous methods with minor modification^12^. Briefly, the mice were individually placed in a cylinder (height: 20 cm, diameter: 10 cm) that was filled with 10 cm of water at 25 ± 1 °C (to avoid a temperature-related stress response) for 6 min. The duration spent immobile was determined by recording the time that the mice stopped struggling and remained floating in the water in an upright position. Moreover, the immobility durations were recorded and analyzed during the last 4 of the 6 min using Noldus EthoVision XT software (Noldus Information Technology Co., Ltd, Netherlands).

The TST was performed in accordance with previously described methods^44^. Briefly, the mice were suspended by a hook 50 cm above soft bedding material in a chamber that was both acoustically and visually isolated. The hook was placed approximately 1 cm from the tip of the tail. The mice were suspended for 6 min, and the immobility duration was recorded and analyzed during the last 4 min of the 6 min test using Noldus EthoVision XT software.

In the sucrose preference test, the animals were trained prior to the operation to consume a 1% sucrose solution. Before each test, they were deprived of food and water for 24 h. The animals were then allowed access to sterile water and a 1% sucrose solution for 1 h. Bottle positions were switched at 0.5 h after the start of the test. Sucrose preference was calculated with the following formula, as described by Willner *et al*.^45^: sucrose preference (%) = (sucrose intake/total fluid intake) × 100%.

### Immunoreactivity quantification

The number of TH-positive DA neurons in the VTA was stereologically estimated. Approximately 32 sections were available for calculation, and every 8^th^ section was examined throughout the entire rostro-caudal extension of the ipsilateral VTA (from approximately -2.92 to -3.88 relative to bregma). The sum of the TH-positive cells was calculated by adding the numbers of TH-positive cells in each selected section. Each experimental group included five mice, and at least four frozen sections were analyzed for each mouse, all of which were assessed in a double-blind manner.

### Statistical analysis

All statistical analyses were performed using SPSS 18.0 software. The data are expressed as the mean ± SEM. Comparisons between 2 groups were analyzed using 2-tailed Student’s t tests. The number of DA neurons, DA concentrations, and Western blot and behavioral data were analyzed using one-way repeated measures ANOVA followed by Scheffe’s post hoc test. A *P* value < 0.05 was considered statistically significant.

### Electronic supplementary material

Supplementary Information
