# Author Correction: Stably maintained microtubules protect dopamine neurons and alleviate depression-like behavior after intracerebral hemorrhage

**DOI:** 10.1038/s41598-024-69192-y

**Published:** 2024-08-08

**Authors:** Yang Yang, Kaiyuan Zhang, Jun Zhong, Ju Wang, Zhongyuan Yu, Xuejiao Lei, Xuezhu Chen, Yulian Quan, Jishu Xian, Yujie Chen, Xin Liu, Hua Feng, Liang Tan

**Affiliations:** 1 Department of Neurosurgery, Southwest Hospital, Third Military Medical University (Army Medical University), 29 Gaotanyan Street, Chongqing, 400038 China; 2 Battalion 3 of Cadet Brigade, Third Military Medical University (Army Medical University), 29 Gaotanyan Street, Chongqing, 400038 China

Correction to: *Scientific Reports* 10.1038/s41598-018-31056-7, published online 23 August 2018

This Article contains errors.

In Figure 3D, the Western blot images for ‘Acetylated tubulin’ and ‘Total tubulin’ are incorrect. The correct Figure 3 and accompanying legend appear below.

**Figure 3:**
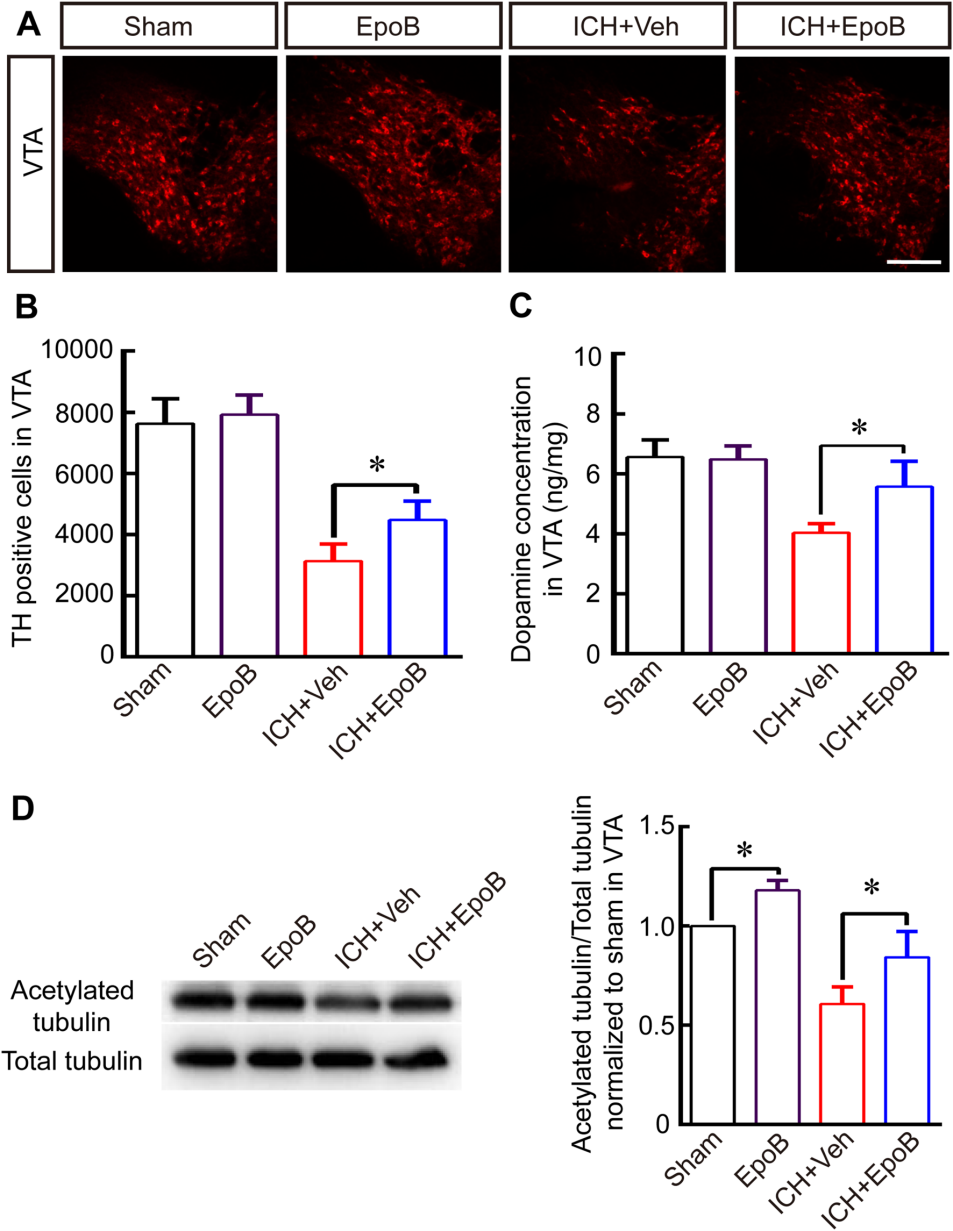
The caption to be typeset alongside it: EpoB treatment rescues DA neurons and DA concentrations by promoting MT stabilization after ICH. (**A**) Representative immunofluorescence staining for TH in VTA in the Sham, EpoB, ICH + Veh and ICH + EpoB groups at 14 days after ICH (Scale bar = 100 μm). (**B**-**C**) Quantitative data for TH-positive cell numbers (**B**) and dopamine concentrations (**C**) in the VTA in the Sham, EpoB, ICH + Veh and ICH + EpoB groups at 14 days post-ICH. (**D**) Representative Western blot images and quantitative data for acetylated α-tubulin and total tubulin expression in the VTA in the four groups at 14 days post-ICH (Full-length blots/gels are presented in Supplementary Figure S2). Data are shown as the means ± SEM (at least n = 5 for each group; *P < 0.05).

In addition, in the Supplementary Information file, the ‘ICH’ and ‘Noco injection’ labels in Supplementary Figure S1 are incorrect.

The correct Supplementary Information file is now linked to this correction notice.

### Supplementary Information

Supplementary Information.

