# Supplementary material for: Author Correction: Stably maintained microtubules protect dopamine neurons and alleviate depression-like behavior after intracerebral hemorrhage
Source: Sci Rep. 2024 Aug 8;14:18427. doi: 10.1038/s41598-024-69192-y (PMC11310519; doi:10.1038/s41598-024-69192-y)
Supplement: Supplementary file 1 — Supplementary Information. [file 41598_2024_69192_MOESM1_ESM.docx]

**Supplementary Figures**

**Stably** **maintained microtubules protect dopamine neurons and alleviate depression-like behavior after intracerebral hemorrhage**

Yang Yang^1^; Kaiyuan Zhang^1^; Jun Zhong^1^; Ju Wang^1^; Xuejiao Lei^1^; Xuezhu Chen^1^; Yulian Quan^1^; Jishu Xian^1^; Yujie Chen^1^; Xin Liu^1^; Hua Feng^1^* and Liang Tan^1^*

1 Department of Neurosurgery, Southwest Hospital, Third Military Medical University (Army Medical University), Chongqing, 29 Gaotanyan Street, 400038, China

*Contract information:

Hua Feng, M.D., Ph.D., Professor or Liang Tan, M.D.

Email address: fenghua8888@vip.163.com or tracy200712@hotmail.com

**Full-length blots used in Fig. 2D**

**
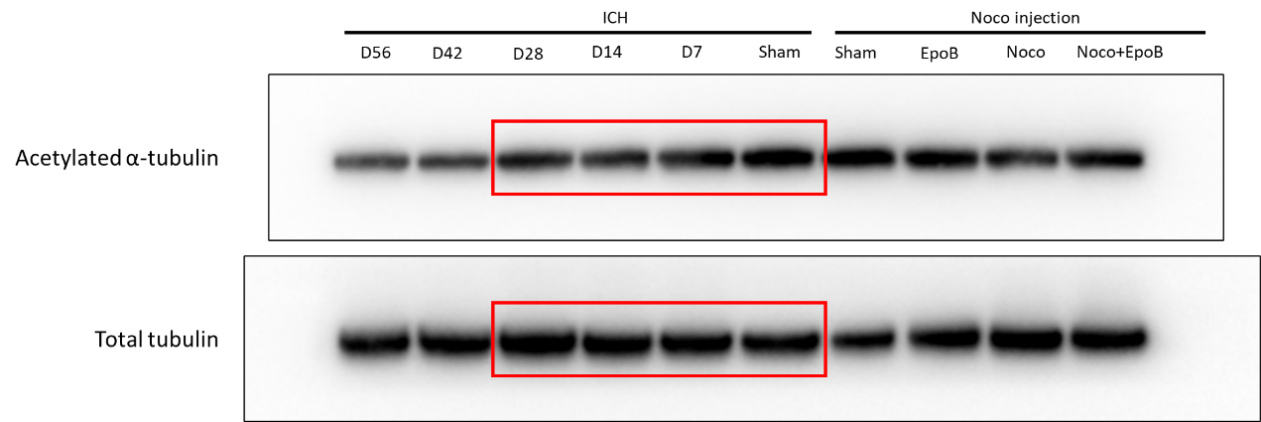
**

**Supplementary Fig. S1. Uncropped images of western blots (1).**

**Full-length blots used in Fig. 3D**


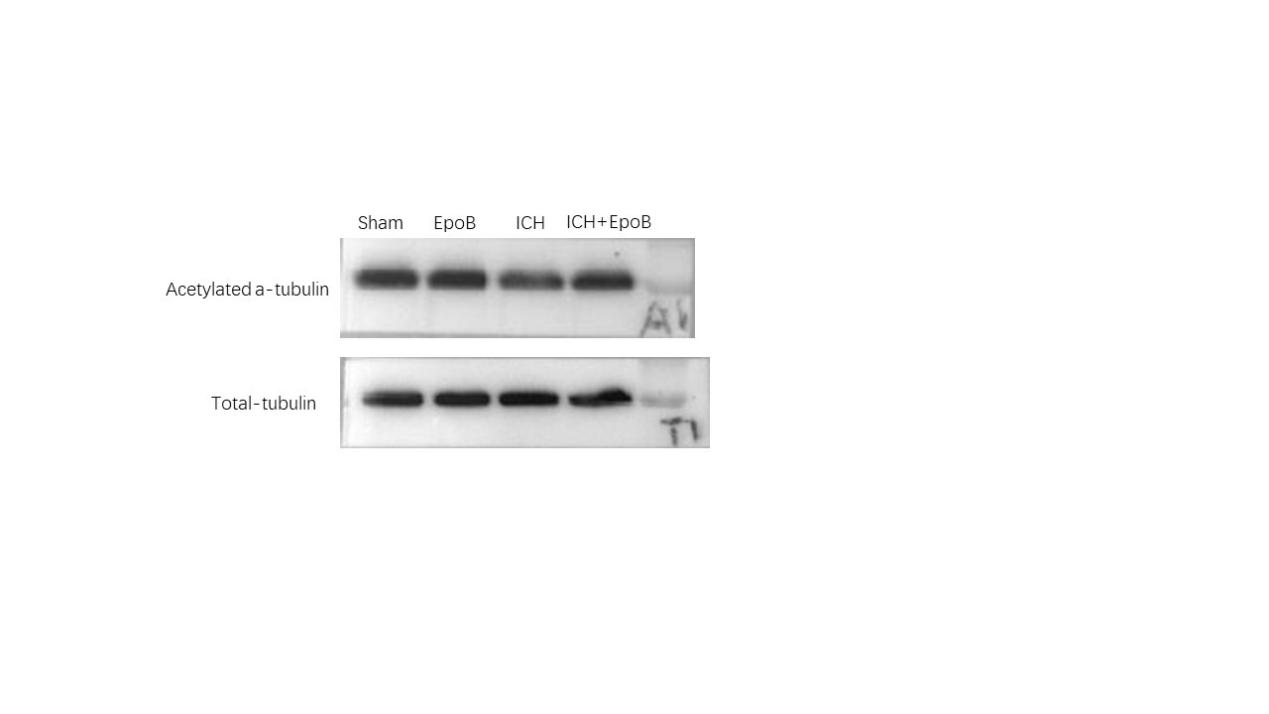


**Supplementary Fig. S2. Uncropped images of western blots (2).**

**Full-length blots used in Fig. 4D**


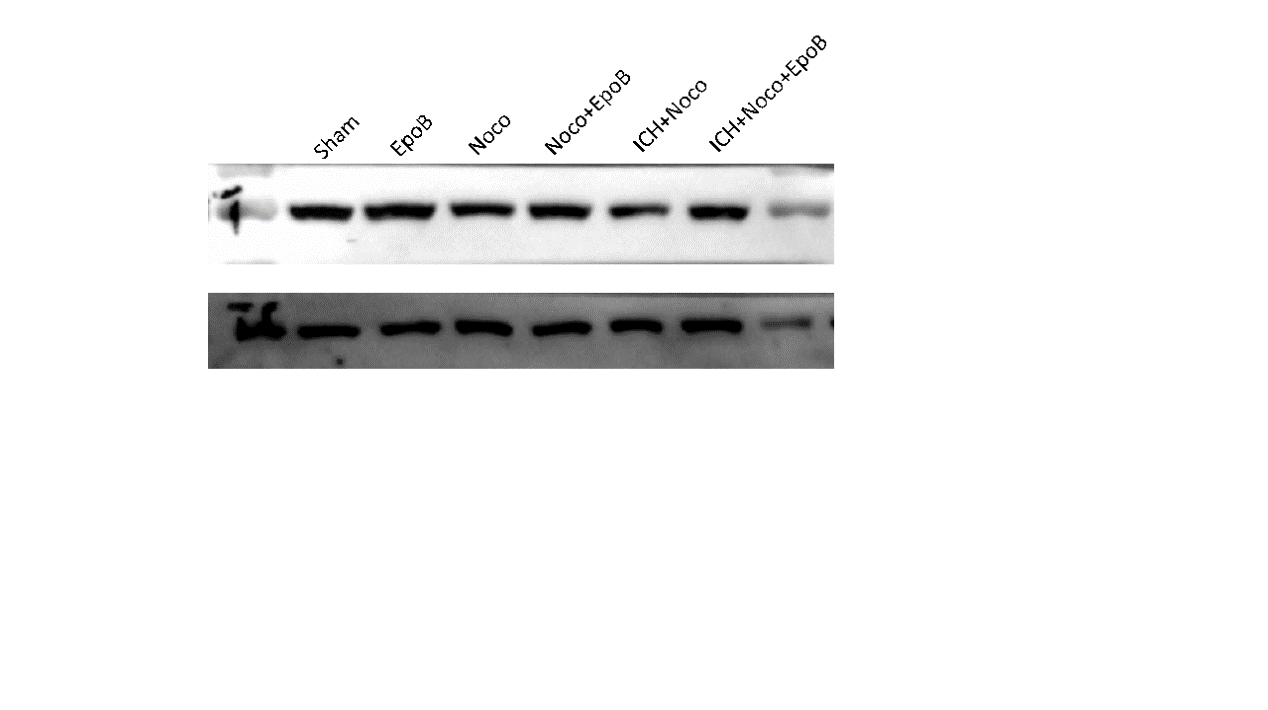


**Supplementary Fig. S3. Uncropped images of western blots (3).**
